# Amputation of Thigh at Lower Third for Destructive Osteo-Arthritis of Knee-Joint, Involving the Lower Part of Femur and Upper End of Tibia

**Published:** 1885-07

**Authors:** A. C. Moreland

**Affiliations:** Atlanta, Ga.


### AMPUTATION OF THIGH AT LOWER THIRD, FOR DESTRUCTIVE OSTEO-ARTHRITIS OF KNEE-JOINT, INVOLVING THE LOWER PART OF FEMUR AND UPPER END OF TIBIA.

---

A CASE FROM THE CLINIC OF THE NEW YORK POLY-CLINIC, SERVICE OF JOHN A. WYETH, M. D., PROFESSOR OF SURGERY, REPORTED BY A. C. MORELAND, M. D., ATLANTA, GA.

---

Reported for the ATLANTA MEDICAL AND SURGICAL JOURNAL.

While the patient was being anæsthetized (ether is used for patients over twelve years of age—chloroform for those under twelve), the following remarks were made by the operator:

This woman is forty years old, native of the United States, single, no children, shirt-maker by trade; her father died at the age of fifty-five, from cancer of the stomach; her mother is sixty-five years old and has phthisis; her sister is in good health; one brother died at thirty-five, from phthisis; a second, at thirteen, from 'congestion of the brain' (probably tubercular meningitis); a third, at twenty-eight, from Bright's disease.

This patient is the only member of her family who has had joint disease; she claims to have been in good health till seven years since, when she was seized with severe pains in her right knee (she denies venereal diseases, rheumatism, cough—everything of this nature), followed by swelling and stiffness of the joint; there is positively no history of injury; the swelling continued for the next few years, the pains being present at varying intervals. Two years ago she was driven to the use of crutches. About this time œdema of the lower extremities appeared, especially marked on the right limb. She came under my care nine days ago; I found the right knee swollen and painful; it measured about three inches more in circumference than its fellow, and there was, as you now observe, a partial sub-luxation of the tibia, backward.

I aspirated the joint, but found no pus or fluid. The history of the case points to tubercular-osteo-arthritis with complete destruction of the joint.

An interesting feature of this case and one which determines the extreme operation of amputation is this—the patient has also chronic nephritis—her urine contains hyaline casts—is of low specific gravity—at times only fourteen ounces are passed in twenty-four hours.

Ordinarily, the patient suffering from no marked visceral lesion, I would open into the joint, gouge away all diseased tissue, establish free drainage, and make an effort to save the leg with anchylosis at the knee. In this case the complication of Bright's disease and the history of phthisis contra-indicate such a procedure.

The patient was covered with blankets and oil clothes, so that all parts of the body were protected excepting the member to be amputated. Esmarch's bandage was applied from the toes to Scarpa's space. In order to avoid the risk of forcing septic matter into the circulation, when the knee was reached the bandage was very loosely applied, being again tightly drawn above the joint. The tourniquet was applied just below Poupart's ligament. Esmarch bandage being removed, the leg was thoroughly washed, first with ether, and then 1-1000 corrosive sublimate solution. All parts not in the field of operation were covered with towels soaked in this solution. Each attendant and the operator disinfected their hands in the same solution. The instruments were submerged into 1-20 carbolic acid solution a half hour before being used. The sponges were washed in 1-1000 bichloride solution.

Antero-posterior symmetrical skin flaps were made. On opening the joint the entire articular surfaces of tibia and femur were found to be destroyed. Section of the femur was made four inches above the knee before healthy bone could be found. All during the operation (at intervals of two or three minutes) the entire wound was irrigated with 1-2500 mercuric chloride solution. The greatest care was taken to arrest all hemorrhage, however small the oozing. Cat-gut ligatures alone were used, these having been immersed for weeks in pure oil of juniper. The flaps were brought together when all bleeding had ceased. Neuber's absorbable bone drains were used, one coming out at each angle of the wound. The interrupted cat-gut suture was used to approximate the flaps. A strip of iodoform gauze was placed over the edges of the wound—over this sublimate gauze two inches thick was extended to the hip—over this was rubber protection. All of this was held in position by sublimate bandage tightly applied to prevent oozing into the dressing. The operation was performed December 18, 1884. On December 29, 1884, the dressing was removed, the drains and sutures had disappeared, the flaps had united throughout the wound, and there was no suppuration.

